# Genetic and Environmental Factors Contributing to Parkinson's Disease: A Case-Control Study in the Cypriot Population

**DOI:** 10.3389/fneur.2019.01047

**Published:** 2019-10-17

**Authors:** Andrea Georgiou, Christiana A. Demetriou, Yiolanda P. Christou, Alexandros Heraclides, Eleni Leonidou, Panayiotis Loukaides, Elena Yiasoumi, Marios Pantziaris, Kleopas A. Kleopa, Savvas S. Papacostas, Maria A. Loizidou, Andreas Hadjisavvas, Eleni Zamba-Papanicolaou

**Affiliations:** ^1^Neurology Clinics, The Cyprus Institute of Neurology and Genetics, Nicosia, Cyprus; ^2^The Cyprus School of Molecular Medicine, The Cyprus Institute of Neurology and Genetics, Nicosia, Cyprus; ^3^Department of Primary Care and Population Health, University of Nicosia Medical School, Nicosia, Cyprus; ^4^Limassol General Hospital, Limassol, Cyprus; ^5^Private Neurology Clinic, Larnaca, Cyprus; ^6^Electron Microscopy and Molecular Pathology Department, The Cyprus Institute of Neurology and Genetics, Nicosia, Cyprus

**Keywords:** Parkinson's disease, environmental factors, genetic variants, Cypriot population, observational study, case-control study, epidemiology

## Abstract

**Introduction:** Parkinson's disease (PD) is a neurodegenerative disorder affecting a substantial proportion of the elderly Cypriot population. The objective of this study was to evaluate PD risk variants that have been identified previously in Genome Wide Association Studies (GWAS) and to find environmental factors that are predictors for PD onset in the Cypriot population.

**Methods:** A case-control study was conducted with a total of 235 PD patients and 464 healthy controls of Greek-Cypriot ethnicity. Demographic and lifestyle characteristics, exposure to PD risk factors and clinical data were collected. Moreover, 13 previously GWAS-identified PD risk variants were genotyped. Univariate and multivariate regression analyses examined the association between a number of environmental and genetic factors and PD.

**Results:** Multivariable regression analysis revealed that exposure to both pesticides and other toxic substances (*P* = 0.03), severe head injury accompanied with fainting (*P* = 0.001), nuts consumption (*P* = 0.004), red meat consumption (*P* = 0.02), and soft drinks consumption (*P* = 0.008) were increasing the risk for PD, whereas cumulative smoking (*P* = 0.02), and fish consumption (*P* = 0.02) were decreasing the risk for PD. Five out of the 13 tested SNPs (rs12185268, rs6599389, rs356220, rs13312, and rs17649553) were confirmed to be nominally significantly associated (*P* < 0.05) with PD risk in the Cypriot population.

**Conclusions:** Collectively, this case-control study has shed some light on the nature of PD epidemiology in Cyprus, by demonstrating a number of genetic and environmental determinants of PD in the Cypriot population.

## Introduction

Parkinson's disease (PD) is a neurodegenerative disorder, characterized by selective loss of dopamine secreting neurons and accumulation of Lewy bodies in the brain and spinal cord (1). It affects 0.3% of the general population and 1% of the population over 60 years old in industrialized countries. The prevalence of the disease is generally higher in Europe and North America compared to South America and Africa (2, 3).

PD is categorized into genetic and sporadic, with the first following Mendelian inheritance and the second being complex. At present, sporadic PD accounts for about 90% of the cases, with the exact pathogenic mechanisms underlying the disease not being completely understood yet (4). However, it is well-known that sporadic PD risk is determined by the complex interplay of genetic and environmental risk factors. Numerous studies and meta-analyses over the last three decades have revealed a number of environmental and genetic risk factors associated with PD risk.

Environmental factors, such as head injury, rural living, pesticides, anxiety/depression, and dairy products intake were positively associated with PD, while physical activity, smoking, coffee consumption, alcohol drinking, smoking, and serum uric acid concentration were reported to be inversely associated with the disease (5, 6).

The genetic component in sporadic PD is currently undisputable. However, the level of heritability of the disease has been debated, with twin studies, family segregation studies and GWAS studies reporting estimates ranging from 6 to 45% (7–9). The heritability value for PD explained by common variants was recently estimated to be 0.21 (10). Currently there are 41 genetic loci that have been associated with PD pathogenesis through 6 large meta-analysis studies (8, 10, 11).

At the moment there is lack of epidemiological data for PD in the Cypriot population. Cyprus is a Mediterranean island and although an isolated population, it is a crossroad between Africa, Europe and Middle East. This made Cyprus a “genetic pool” for transiting populations which gave the genetic signature to the Cypriot population today, characterized by genetic affinity with surrounding Southeast European and Near Eastern populations (12). This renders genetic studies in the Cypriot population informative for the genetically similar populations as well. Characteristic of the genetic admixture and of the peculiarity of the Cypriot population are the geographical clusters of other neurological genetic diseases such as Friedreich ataxia, Huntington disease, and Familial Amyloid Polyneuropathy (13–15). Therefore, the investigation of the epidemiology of other neurological diseases such as PD in the Cypriot population is of particular interest. In addition, it is interesting to investigate which environmental factors are associated with PD in the Cypriot population, a population where some of the PD risk factors are of high prevalence and compare the findings with similar studies involving different populations.

Herein, we aimed to investigate both genetic and environmental determinants of PD in the Cypriot population. Previously published work by our group showed that mitochondrial haplogroups influence the PD risk and age of onset in a gender-specific manner (16). This is the first study exploring the epidemiology of PD in the Cypriot population and will function as a baseline for future studies concerning the etiology as well as the early diagnosis of PD.

## Methods

### Study Population and Exposure Assessment

A cohort of 235 PD patients and 464 control subjects were recruited from multiple medical and community centers across Cyprus as described previously (16). Patients were included in the study after clinical diagnosis of PD by a board certified neurologist. Diagnosis was followed by a clinical evaluation, using the UPDRS rating scale by a board certified CING neurologist. Patients that had clinical signs suggestive of Parkinsonian syndromes were excluded.

The 464 ethnically-matched controls were recruited using random cluster sampling across all the districts of Cyprus. Cluster sampling included mailing letters of invitation to residences in randomly selected postal codes as well as visiting randomly selected medical/community centers across Cyprus. Individuals that were ≥45 years old and did not suffer from any neurodegenerative disorder or cognitive impairment were invited to participate as controls. All study participants were of Greek-Cypriot nationality.

Epidemiological data from all study participants were collected through a personal interview. The questionnaires consisted of five main sections, which were assessed retrospectively: demographic data, environmental exposure to factors that associated with PD in previous studies (exposure to pesticides and other toxic agents, well water consumption, severe head injury, and intense stress), medical history, lifestyle (diet habits, smoking, alcohol consumption), and anthropometric data (BMI) (5, 6). The questionnaire addressed to the patients, had an additional section covering information about the age of onset, the type of the disease and the symptoms of the disease for each patient.

### SNP Selection and Genotyping

Thirteen SNPs that have been associated with PD (*p* ≤ 5 × 10^−8^) in at least one out of the 5 large GWAS meta-analysis studies for PD in the European population were selected for genotyping (Supplementary Table 1) (10, 11, 17–19). The selection criteria for the SNPs were based on the estimates of the association (0.81 > OR > 1.23) and on the frequency of the minor allele (MAF > 5%), in order to ensure the maximum statistical power for their investigation. There was an estimation of the power of the study at a value of 0.05 to detect ORs similar to those previously reported in the GWAS, given the allele frequencies observed in the Cypriot population.

DNA was extracted from peripheral blood lymphocytes as described elsewhere (14). SNP genotyping was performed using Taqman genotype assays (Thermo Fisher Scientific). Each assay was carried out using 10 ng genomic DNA in a 5 μl reaction using Taqman Universal PCR Master Mix (ABI). The fluorescence profile was read on an ABI PRISM 7900HT instrument and the results analyzed with Sequence Detection Software (ABI).

### Statistical Analysis

Statistical analysis was separated into four parts: descriptive analysis of demographic data, univariate logistic regression analysis, multi-variable logistic regression analysis, and logistic regression for the genetic analysis.

Demographic characteristics of cases and controls were described as frequency and percentage for categorical variables and median and interquartile range (IQR) for continuous variables with a non-normal distribution.

For the comparison of numerical variables between cases and controls the non-parametric Mann Whitney Wilcoxon test was used. For the categorical variables, the chi-square test was employed to compare the frequencies of cases and controls.

Univariate non-adjusted logistic regression analysis was used to test for any association between each variable and PD status. The exposure variables were separated into two large categories: lifestyle characteristics and previously reported exposure risk factors. Lifestyle risk factors included cumulative smoking (cigarettes over lifetime), coffee consumption (cups per month), alcohol intake (glasses per month), food dietary habits (frequency of consumption per month), and indoor and outdoor activities (hours spent per week). Six food categories that are over-represented in the Mediterranean Diet were chosen to construct a new variable called “healthy eating.” The Kruskal Wallis non-parametric test was carried out to test whether age of onset differed between the different food consumption categories. Previously reported exposure risk factors include exposure to pesticides, exposure to other toxic and chemical substances, well water drinking, previous severe head injury and exposure to a traumatic experience.

Following all binary logistic regression analyses, the significantly predicting PD risk factors were combined into a multi-variable logistic regression model. Bonferroni correction was applied to account for multiple testing. This enabled us to assess and adjust simultaneously for multiple covariates in relation to a dichotomous outcome; in this case PD.

Trend test was performed for categorical or categorized variables to test if there was a dose-response function between the exposure and the outcome. The level of statistical significance value for the trend analysis test was the 0.05.

All statistical analyses concerning the environmental risk factors were performed using STATA V12 SE statistical software package. SNPStats web-based application (http://bioinfo.iconcologia.net/SNPstats) was used for descriptive statistics of SNPs and assessment of the association of each SNP with PD. Statistical analysis included logistic regression models, adjusted for the age and gender of participants. The log additive model—which indicates how the risk for the disease is modified by each additional minor allele—was chosen to test the association for each SNP with PD.

## Results

### Descriptive Analysis of Demographic Data

A total of 235 PD cases (mean age 66.5 ± 10.5 years, mean age-of-onset 60.4 ± 11.4 years, 54.5% males and 45.5% females) and 464 controls (mean age 65 ± 10.7 years, 50% males and 50% females) were enrolled in this study. PD cases were classified into tremor-dominant (84%) and non-tremor dominant (16%). The prevalence of the most common PD motor and non-motor symptoms of PD cases and their corresponding age at onset are shown in Supplementary Figure 1. The demographic characteristics of the study population are listed in Table 1 and Supplementary Table 2. Mann Whitney Wilcoxon test showed that there was a statistically significant difference between the current age of the two groups (*p* < 0.0001), while there was also a significant difference between the age at onset of PD cases and age at recruitment of controls (*p* < 0.0001). Chi square test revealed a statistically significant difference between PD cases and controls for retirement status and BMI (*p* < 0.0001). Logistic regression revealed that BMI was inversely associated with PD, while retirement was positively associated with PD risk after adjusting for current age (Supplementary Tables 3, 4).

**Table 1:** Demographic characteristics of Cypriot PD cases and controls.

**Variable**		**Total**	**Cases**	**Controls**	*p*-value^*^ (test)
**Current age**	*N*	691	229	462	**<0.0001** (Wilcoxon)
	Median (IQR)	67 (17)	70 (12)	64.5 (16)	
**Age at baseline**	*N*	685	226	455	**<0.0001** (Wilcoxon)
	Median (IQR)	64 (15)	62 (16)	64.5 (16)	
**No of children**	*N*	691	229	462	0.87 (Wilcoxon)
	Median (IQR)	3 (1)	3 (1)	3 (1)	
**Gender**					
Male	*N* (%)	358 (51.5)	127 (54.5)	231 (50.0)	0.26 (chi-square)
Female	*N* (%)	337 (48.5)	106 (45.5)	231 (50.0)	
**BMI (kg)**					
Normal weight 20–24.9	*N* (%)	165 (27.3)	64 (34.0)	101 (24.3)	**0.01** (chi-square)
Underweight ≤20	*N* (%)	21 (3.5)	13 (6.9)	8 (1.9)	
Overweight 25–29.9	*N* (%)	252 (41.7)	78 (41.5)	174 (41.8)	
Obesity >30	*N* (%)	166 (27.5)	33 (17.6)	133 (32.0)	
**Education level**					
Primary school	*N* (%)	281 (40.5)	104 (45.4)	177 (38.0)	0.13 (chi-square)
Secondary school	*N* (%)	94 (13.5)	23 (10.0)	71 (15.3)	
High school	*N* (%)	199 (28.7)	61 (26.6)	138 (29.7)	
Bachelor's degree or higher	*N* (%)	120 (17.3)	41 (17.9)	79 (17.0)	
**Retirement**					
Not yet	*N* (%)	198 (30.8)	30 (14.1)	168 (39.2)	**<0.001** (chi-square)
Yes	*N* (%)	320 (49.8)	113 (53.1)	207 (48.3)	
Yes, early	*N* (%)	124 (19.3)	70 (32.9)	54 (12.6)	

* *P-value nominal significance threshold = 0.05*.

*Significant p-values are marked in bold*.

### Univariate Logistic Regression Analysis

Smoking, coffee consumption, alcohol consumption and food dietary habits were tested for their association with PD risk using univariate logistic regression analysis (Table 2 and Supplementary Table 5). There was statistically significant evidence that heavy smokers had about two times less risk to develop PD than non-smokers (OR: 0.45, 95% CI: 0.23–0.91). Coffee consumption was also a predictor for PD in the Cypriot population, with those in the lowest quartile of coffee consumption exhibiting a double risk for PD than participants in the highest quartile (OR: 0.53, 95% CI: 0.33–0.85). This coffee consumption—PD risk inverse association survived Bonferroni correction. Although there was no significant evidence to support that total alcohol consumption affects the risk for PD, heavy wine consumption was inversely associated with PD risk, without accounting for any confounders (OR: 0.54, 95% CI: 0.30–0.96) (Supplementary Table 5).

**Table 2:** Lifestyle and previously reported exposure risk factors in Cypriot PD cases and controls.

**Variable**		**Total**	**Cases**	**Controls**	OR^*^ (95% CI)	*p*-value^**^ (LR)	*p*-trend^***^ (LR)
**CUMULATIVE SMOKING (CIGARETTES OVER LIFETIME)**							
Q0: 0	*N* (%)	377 (58.9)	138 (65.4)	239 (55.7)	1.00		**0.02**
Q1: 1–48,000	*N* (%)	53 (8.3)	14 (6.6)	39 (9.1)	0.62 (0.33–1.19)	0.15	
Q2: 48,000–132,000	*N* (%)	50 (7.8)	17 (8.1)	33 (7.7)	0.89 (0.48–1.66)	0.72	
Q3: 132,000–275,000	*N* (%)	53 (8.3)	16 (7.6)	37 (8.6)	0.75 (0.40–1.40)	0.36	
Q4: 275,000–438,000	*N* (%)	54 (8.4)	15 (7.1)	39 (9.1)	0.67 (0.35–1.25)	0.21	
Q5: 438,000–1,940,000	*N* (%)	53 (8.3)	11 (5.2)	42 (9.8)	0.45 (0.23–0.91)	**0.026**	
**TOTAL COFFEE CONSUMPTION (CUPS PER MONTH)**							
Q1: 0–28	*N* (%)	187 (27.5)	77 (34.1)	110 (24.3)	1.00		**0.009**
Q2: 28–56	*N* (%)	180 (26.5)	57 (25.2)	123 (27.2)	0.66 (0.43–1.02)	0.06	
Q3: 56–84	*N* (%)	168 (24.7)	53 (23.5)	115 (25.4)	0.66 (0.43–1.02)	0.06	
Q4: 84–420	*N* (%)	144 (21.2)	39 (17.3)	105 (23.2)	0.53 (0.33–0.85)	**0.008**	
**TOTAL ALCOHOL (GLASSES PER MONTH)**							
0	*N* (%)	234 (33.3)	77 (33.0)	153 (32.8)	1.00		0.65
Q1: 0–2.5	*N* (%)	118 (16.8)	43 (18.4)	75 (16.1)	1.14 (0.72–1.81)	0.58	
Q2: 2.5–11.4	*N* (%)	117 (16.6)	36 (15.5)	81 (17.4)	0.88 (0.55–1.43)	0.61	
Q3: 11.4–33.4	*N* (%)	117 (16.6)	41 (17.6)	76 (16.3)	1.07 (0.67–1.71)	0.67	
Q4: 33.4–496	*N* (%)	117 (16.6)	36 (15.5)	81 (17.4)	0.88 (0.55–1.43)	0.61	
**HEALTHY EATING**							
0	*N* (%)	32 (4.6)	17 (7.3)	11 (2.4)	1		**0.03**
1	*N* (%)	60 (8.5)	18 (7.7)	42 (9.0)	0.28 (0.11–0.71)	0.007^*^	
2	*N* (%)	102 (14.5)	36 (15.5)	66 (14.2)	0.35 (0.15–0.83)	0.02^*^	
3	*N* (%)	142 (20.20)	51 (21.9)	91 (19.5)	0.36 (0.16–0.83)	0.02^*^	
4	*N* (%)	188 (26.7)	58 (24.9)	130 (27.9)	0.29 (0.13–0.65)	0.003^*^	
5	*N* (%)	138 (19.6)	41 (17.6)	97 (20.8)	0.27 (0.12–0.63)	0.003^*^	
6	*N* (%)	41 (5.8)	12 (5.2)	29 (6.2)	0.27 (0.10–0.74)	0.01^*^	

*LR, Logistic Regression analysis*.

* *Univariate non-adjusted Logistic Regression Model*.

** *P-value nominal significance threshold = 0.05*.

*** *Bonferroni adjusted significance threshold = 0.01*.

*Significant p-values are marked in bold*.

Considering dietary habits, PD cases were consuming significantly more nuts, olives, red meat, carbohydrate rich food, and soft drinks than controls. However, fish consumption was significantly lower in PD cases than controls. PD cases had a significantly lower adherence to “healthy eating” when compared to controls. The associations between food categories and PD risk that remained statistically significant after Bonferroni correction were the following: nuts-PD, red meat-PD, soft drinks-PD and healthy eating-PD. Kruskal Wallis test showed that there was significant difference at the age of onset of PD in Cypriot cases depending on “healthy eating” variable (*p* = 0.025) (Supplementary Figure 2). Physical activity was recorded as indoor and outdoor activities. However, no association was observed between physical activity duration and risk for PD (Supplementary Table 6).

Given the positive association previously found between the exposure to pesticides or other chemical substances and PD risk, we evaluated this relationship in the Cypriot population (Table 3 and Supplementary Table 7). Study participants that were exposed to chemical agents had a 64% increased risk for PD (OR: 1.64, 95% CI: 0.99–2.71). The association was considerably stronger when the participants were exposed to pesticides in addition to chemical agents (OR: 3.5, 95% CI: 1.57–7.79). Severe head injury with fainting was also positively associated with PD risk (OR: 1.94, 95% CI: 1.28–2.94). There was evidence supporting that moderate traumatic experience was associated with a 47% decreased risk for PD (OR: 0.53, 95% CI: 0.35–0.82).

**Table 3:** Previously reported exposure risk factors in Cypriot PD cases and controls.

**Variable**		**Total**	**Cases**	**Controls**	OR^*^ (95% CI)	*p*-value^**^ (LR)	*p*-trend^***^ (LR)
**TOXIC AGENTS**							
No toxic agents	*N* (%)	368 (53.3)	108 (47.6)	260 (56.2)	1		**0.001**
Pesticides	*N* (%)	216 (31.3)	71 (31.3)	145 (31.3)	1.18 (0.82–1.7)	0.37	
Other chemical agents	*N* (%)	79 (11.5)	32 (14.1)	47 (10.2)	1.64 (0.99–2.71)	**0.05**	
Both	*N* (%)	27 (3.9)	16 (7.1)	11 (2.4)	3.50 (1.57–7.79)	**0.002**	
**WELL WATER CONSUMPTION**							
No	*N* (%)	297 (43.2)	95 (42.0)	202 (43.8)	1		0.17
Yes, rarely	*N* (%)	104 (15.1)	24 (10.6)	80 (17.4)	0.64 (0.38–1.07)	0.09	
Yes, systematically	*N* (%)	286 (41.6)	107 (47.4)	179 (38.8)	1.27 (0.90–1.79)	0.17	
**SEVERE HEAD INJURY**							
No	*N* (%)	489 (71.0)	144 (63.7)	345 (74.5)	1		**0.03**
Yes, with no fainting	*N* (%)	86 (12.5)	31 (13.7)	55 (11.9)	1.35 (0.83–2.18)	0.22	
Yes, with fainting	*N* (%)	114 (87.5)	51 (22.6)	63 (13.6)	1.94 (1.28–2.94)	**0.002**	
**INTENSE STRESS/TRAUMATIC EXPERIENCE**							
No	*N* (%)	215 (31.5)	79 (35.6)	136 (29.6)	1		0.96
Yes, moderate	*N* (%)	195 (28.6)	46 (20.7)	149 (32.4)	0.53 (0.35-0.82)	**0.004**	
Yes, severe	*N* (%)	272 (39.9)	97 (43.7)	175 (38.0)	0.95 (0.66-1.38)	0.81	

*LR, Logistic Regression analysis*.

* *Univariate non-adjusted Logistic Regression Model*.

** *P-value nominal significance threshold = 0.05*.

*** *Bonferroni adjusted significance threshold = 0.01*.

*Significant p-values are marked in bold*.

### Multi-Variable Logistic Regression Analysis

**Figure 1:**
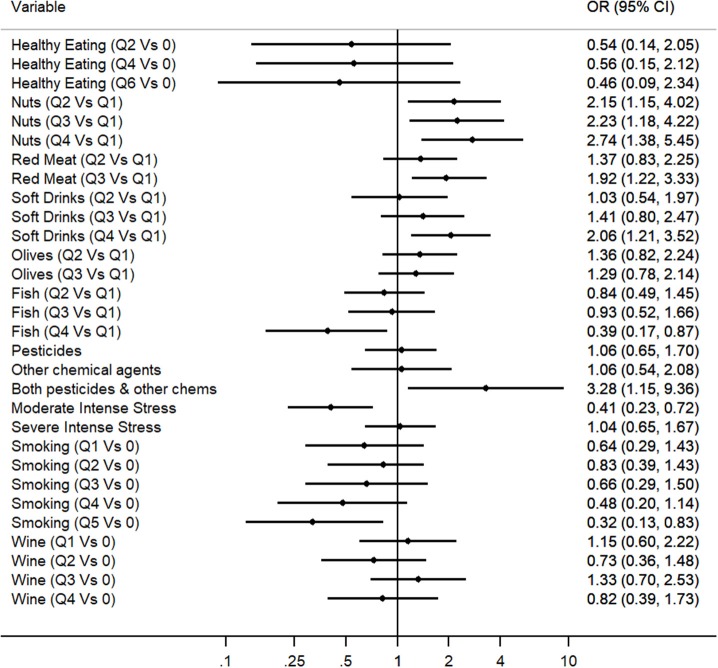
Multivariate logistic regression analysis for the evaluation of the association between environmental factors and PD (OR and 95%CI are represented for each environmental factor).

Multivariable Regression Analysis was applied to explore which of the identified predictors for PD in the Cypriot population were independently associated with the disease, even after the adjustment for possible confounders. Therefore, the predictive multivariable model included all 12 variables that exhibited a nominally significant association with PD risk within the unadjusted regression analysis, excluding coffee consumption due to its high collinearity with smoking and adding the age at baseline and gender variables as covariates (Figure 1 and Supplementary Table 8). Multivariate logistic regression Model 1 revealed that the following variables were predictors for PD: fish consumption (OR: 0.39, 95% CI: 0.17–0.87), nuts consumption (OR: 2.74, 95% CI: 1.38–5.45), red meat consumption (OR: 1.92, 95% CI: 1.22–3.33), soft drinks consumption (OR: 2.06, 95% CI: 1.21–3.52), exposure to both pesticides and other toxic substances (OR: 3.28, 95% CI: 1.15–9.36), severe head injury with fainting (OR: 2.42, 95% CI: 1.43–4.09), moderate traumatic experience (OR: 0.41, 95% CI: 0.23–0.72), and heavy smoking (OR: 0.32, 95% CI: 0.13–0.83). Olive consumption, healthy eating, and heavy wine consumption did not preserve nominally statistical association within the multivariate analysis.

### Genetic Analysis

Supplementary Tables 9, 10 illustrate the allele and genotype frequencies, respectively, in PD cases and controls, for the 13 SNPs evaluated as well as the allele frequencies for each SNP as reported in the 1000 Genomes project. Deviation from Hardy Weinberg equilibrium was not observed for any of the SNPs in the control subjects (*P* = 0.15–1). Five out of the 13 SNPs (rs12185268 (OR: 0.69, 95% CI: 0.52–0.90), rs6599389 (OR: 1.50, 95% CI: 1.04–2.16), rs356220 (OR: 1.33, 95% CI: 1.05–1.67), rs13312 (OR: 1.68, 95% CI: 1.23–2.28), and rs17649553 (OR: 0.71, 95% CI: 0.54–0.93) were statistically significantly associated with PD in this study at P less than 0.05 (Table 4). Rs12185268 is a missense variant located in *SPPL2C* gene, while rs13312 is a non-coding variant located in the 3 prime untranslated region of *USP24* gene. Rs6599389, rs356220, and rs17649553 are intron variants located in *TMEM175, SNCA*, and *MAPT* genes, respectively (http://www.ncbi.nlm.nih.gov/SNP/). Rs823118 (OR: 0.79, 95% CI: 0.62–1.01) and rs356182 (OR: 1.24, 95% CI: 0.98–1.57) SNPs marginally missed the nominal significance level for association with PD risk. The direction of the association of the seven SNPs with PD in the Cypriot population was in line with the direction of the association described in previous GWAS studies.

**Table 4:** OR and 95% CI for the associations between 13 SNPs and PD risk.

**#**	**SNP**	**Minor allele**	OR (95% CI)^*^	*P*-value^**^
1	rs12185268	G	0.69 (0.52–0.90)	**0.006^∧^**
2	rs10513789	G	1.09 (0.82–1.45)	0.57
3	rs6599389	A	1.50 (1.04–2.16)	**0.03**
4	rs356220	T	1.33 (1.05–1.67)	**0.02**
5	rs7617877	A	1.03 (0.80–1.34)	0.80
6	rs17115100	T	1.06 (0.74–1.53)	0.75
7	rs10464059	A	1.13 (0.80–1.60)	0.49
8	rs13312	G	1.68 (1.23–2.28)	**0.001**
9	rs1801582	G	1.08 (0.80–1.46)	0.63
10	rs4837628	C	0.89 (0.69–1.14)	0.36
11	rs823118	C	0.79 (0.62–1.01)	0.056
12	rs356182	G	1.24 (0.98–1.57)	0.076
13	rs17649553	T	0.71 (0.54–0.93)	**0.013**

* *Logistic Regression Model adjusted for age and gender*.

** *P-value Nominal significance threshold = 0.05*.

∧ *Bonferroni adjusted significance threshold = 0.004*.

*Significant p-values are marked in bold*.

## Discussion

This case-control study confirmed for the first time a number of predictors for PD, related to environmental exposure and genetic risk factors, for the Cypriot population.

The proportion of PD cases that retired early (<65 years old) was almost three times larger than the proportion of controls that retired early. This was in line with an observational cohort study for PD, which showed a hazard ratio of 2.08 for an earlier retirement associated with PD status (20). This shows that the ability to remain in the workforce decreases significantly as the time since onset of the disease increases. Motor and non-motor symptoms make holding an occupation challenging in many psychological and biological aspects for PD patients.

The role of BMI in PD risk is still uncertain, with conflicting results by different epidemiological studies (21, 22). In the current study, we observed a significant inverse association between BMI with PD risk. Weight loss is a frequent early PD symptom as a result of gastrointestinal dysfunction and anorexia (23). In some cases, nutritional complications pre-exist motor-related symptoms. Therefore, one logical interpretation for the inverse association between BMI and PD could be reverse causation. However, this finding is in line with a recent Mendelian randomization study that found a causal association between lifetime exposure to higher BMI and a lower risk for PD (24).

There is compelling evidence that both smoking and coffee consumption are inversely associated with PD risk (25). Our univariate findings regarding cumulative smoking and PD and coffee consumption and PD are consistent with previous findings reporting a protective effect of smoking and coffee consumption for PD. Cumulative smoking was still significantly protective for PD onset when the regression analysis was adjusted for multiple variables. This finding lends support to the hypothesis that biological mechanisms are involved in the smoking-PD relation. One such possible mechanism is the neuroprotective effect of nicotine by modulating the activity of mitochondrial complex I of the respiratory chain and by activating nicotinic acetylcholine receptors in dopaminergic neurons (26).

In the present study, we observed that PD patients had different dietary habits than controls. The multivariable analysis revealed fish consumption as a protective factor and red meat, nuts, and soft drinks consumption as risk factors for the onset of PD. A significant reduction of fish consumption among PD cases was also observed in another retrospective study in the Italian population (27). This protective association is supported by a rat model study proposing that a combination of fish oil with other neuroprotective substances is likely to provide a superior therapeutic advantage in the prevention of oxidative stress-mediated neurodegenerative conditions such as PD (28). This is the first study detecting an increased risk for PD for moderate and heavy soft drinks consumers. A possible explanation could be given by a rat model study which demonstrated that carbonated soft drinks induced oxidative stress and also altered the expression of certain genes associated with brain activity (29). However, soft drinks cover a broad range of drinks, with a large number of components, making it challenging to trace the component that could potentially cause neurodegeneration. The fact that nuts were positively associated with PD risk in the present study could be attributed to the fact that nuts are rich in manganese and iron. The high dietary intake of both iron and manganese demonstrated an almost 2 fold higher risk for PD elsewhere (30). Also nuts have high levels of proteins and fat where organochlorine pesticides are accumulated as it was shown in a toxicology study carried out in India (31). Pesticides inside nuts can accumulate not only from direct pesticide application but also from pesticides concentrated in the soil where nut trees grow. The significant positive association between red meat and PD risk, may be explained by the heme content that may act as a toxin when not digested properly (32). Although there is no study reporting any significant association between red meat by itself and PD risk, it was demonstrated that high intake of animal fat accompanied with low transferrin saturation levels exhibited a 9 fold increased risk for PD when compared to low animal fat intake (33). In addition red meat is rich in saturated fats which increase oxidative stress (34). An unexpected positive association was detected between olives and PD in the univariate analysis. However, this association faded away after adjusting for current age of participants. This can be explained by the fact that older Cypriots tend to consume olives more frequently than younger Cypriots, thus rendering age as confounder in the association.

Exposure to both pesticides and chemical agents were positively associated with PD risk in this case-control study, being consistent with the findings of previous studies (35). One possible interpretation for this positive association could be that the exposure to a variety of environmental toxicants, including pesticides has been associated with differential DNA methylation of genes encoding for enzymes which are key players in cellular redox homeostasis which was found to be involved in PD pathogenesis (36). The results regarding severe head injury with fainting are similar to the pooled results of a meta-analysis study that included 22 studies testing the association between head injury and PD risk (37). Surprisingly, there was a statistically significant protective association detected between moderate intense stress and PD risk. This is possibly a false positive result which could be attributed to the fact that what a PD patient considers as a moderate intense stress differs from what a healthy control considers as a moderate intense stress after the shock of PD diagnosis.

Recent genome wide meta-analysis studies have identified several susceptibility loci for PD (10, 11, 17–19). We have replicated the association of 5 previously reported common variants of small effect size within the *SPPL2C, TMEM175, SNCA, USP24*, and *MAPT* loci for the Cypriot population, even though the analysis was underpowered (8, 11). The significance of the detected associations between the genotyped SNPs and PD risk were weaker in this study when compared to other larger studies from different populations (8, 11, 38). There are two possible scenarios for the failure to replicate the association for the remaining genetic variants. The first explanation could be the restricted power of our study to detect associations with variants of small effect size due to the small sample size and the second could be the fact that the genetic variants identified in previous GWA studies are just proxies for the putative functional variants and therefore population-specific differences allele frequencies and in linkage disequilibrium patterns.

This is the first study exploring both the genetic and environmental determinants for PD in the Cypriot population. Therefore, the results of the current study shed some light regarding understanding the nature of PD epidemiology in the Cypriot population. In addition, given the fact that a large proportion of Cypriots were exposed to risk factors such as pesticides, well water consumption, and intense stress (due to the 1974 war) renders the study essential in understanding which of this factors increase PD in this population and in devising the appropriate prevention strategies. However, the current study has some limitations, including its small sample size which leads to low study power being perhaps its greatest restriction. Despite, the sample size is generally adequate for very common exposures; it did not provide sufficient power for the detection of expected associations for rare exposures. However, given the fact that Cyprus is a small country and the fact that PD is not a common disease, although it affects a considerable proportion of the elderly population, a larger sample size was almost impossible to recruit. Lastly, due to the observational nature of this study, no inferences could be made regarding the causal nature of the associations identified.

In conclusion, the current study has demonstrated a number of genetic and environmental predictors for PD in the Cypriot population. Multivariable regression analysis revealed that exposure to both pesticides and other toxic substances, severe head injury accompanied with fainting, nuts consumption, red meat consumption, and soft drinks consumption were predisposing factors, whereas cumulative smoking and fish consumption were protective factors for PD risk. The association between rs12185268, rs6599389, rs356220, rs13312, and rs17649553 SNPs and PD risk was replicated in the Cypriot population.

## Data Availability Statement

Access to the source data used in this study are available through: https://www.ebi.ac.uk/eva/?eva-study=PRJEB32182.

## Ethics Statement

The study was carried out in accordance with the recommendations of the Cyprus National Bioethics Committee. The protocol was approved by the Cyprus National Bioethics Committee. All subjects gave written informed consent in accordance with the Declaration of Helsinki.

## Author Contributions

AG: (1). Research Project A. Conception, B. Organization, C. Execution, (2). Statistical Analysis A. Design, B. Execution, (3). Manuscript A. Writing of the first draft. CD: (1). Research Project A. Conception, B. Execution, (2). Statistical Analysis A. Design, B. Review and Critique, (3). Manuscript A. Review and Critique. YC and AHe: (1). Research Project A. Conception, B. Execution. EL, PL, EY, MP, KK, and SP: (1). Research Project A. Execution. ML and AHa: (1). Research Project A. Execution. (2). Manuscript A. Review and Critique. EZ-P: (1). Research Project A. Conception, (2). Manuscript A. Review and Critique.

### Conflict of Interest

The authors declare that the research was conducted in the absence of any commercial or financial relationships that could be construed as a potential conflict of interest.
